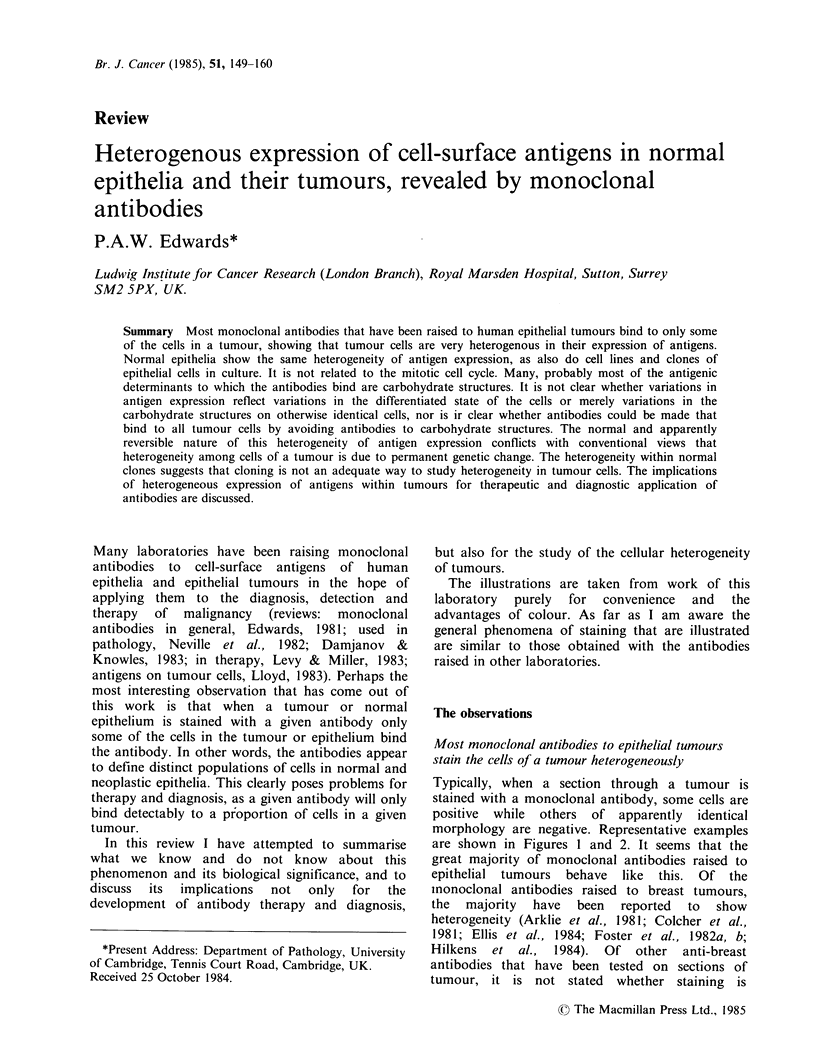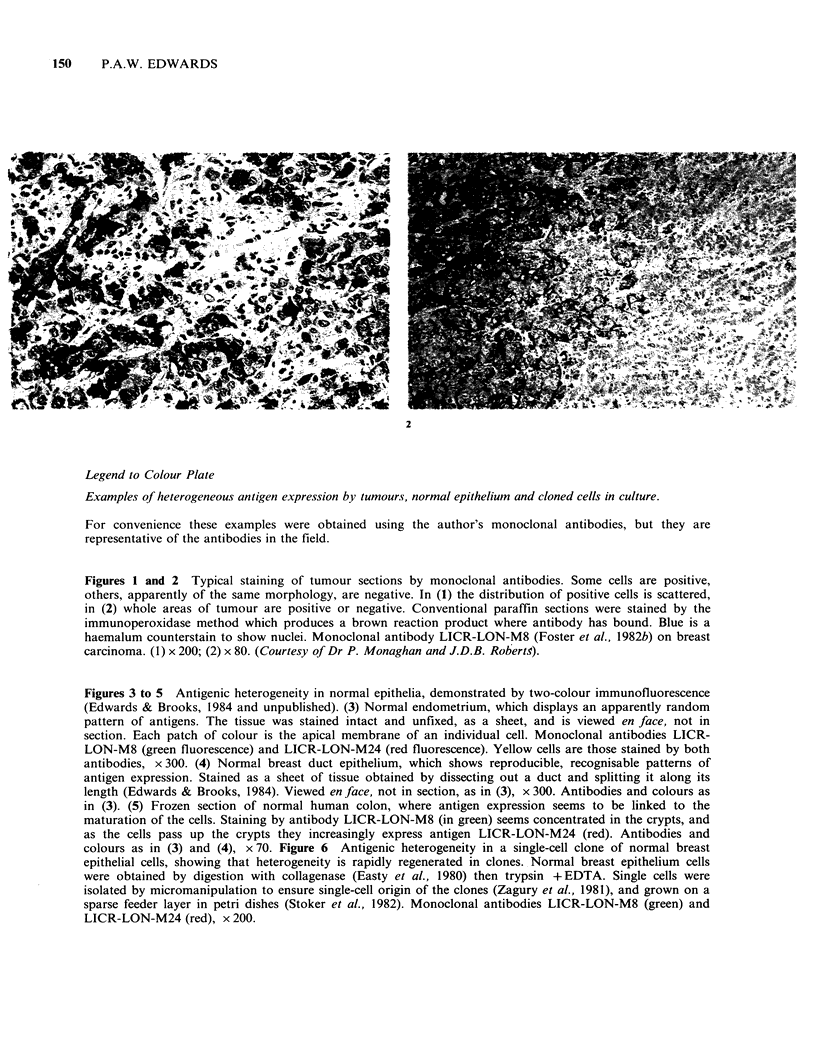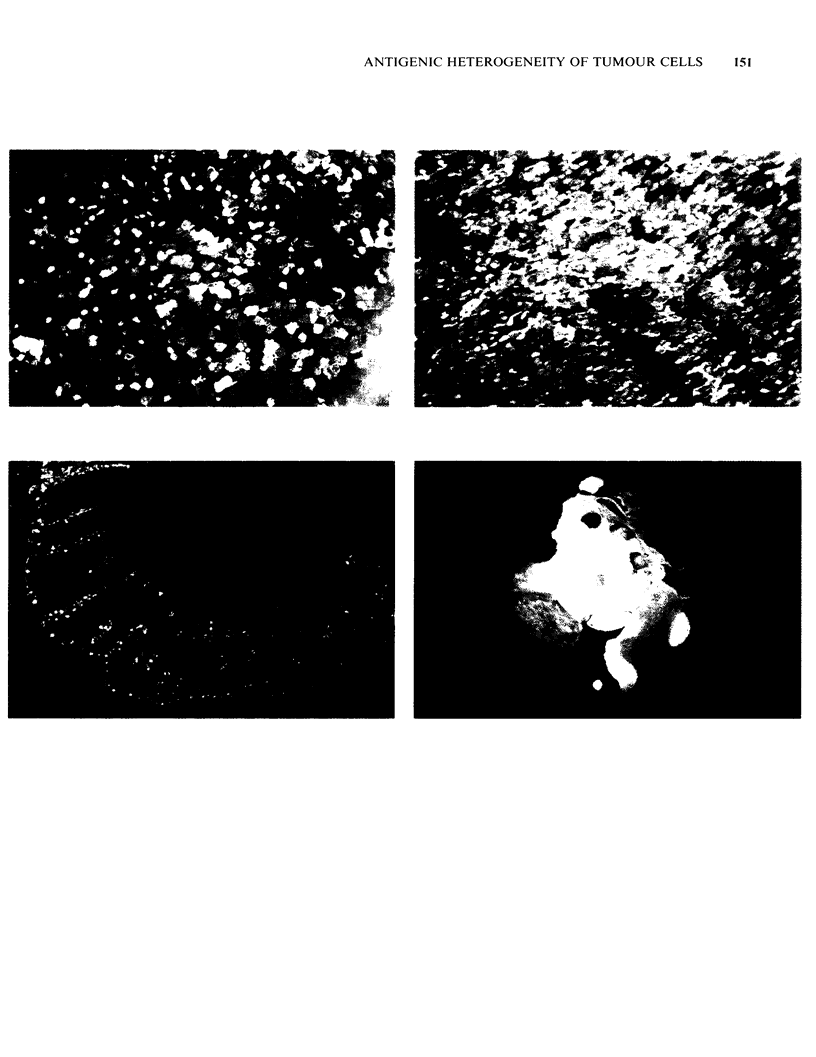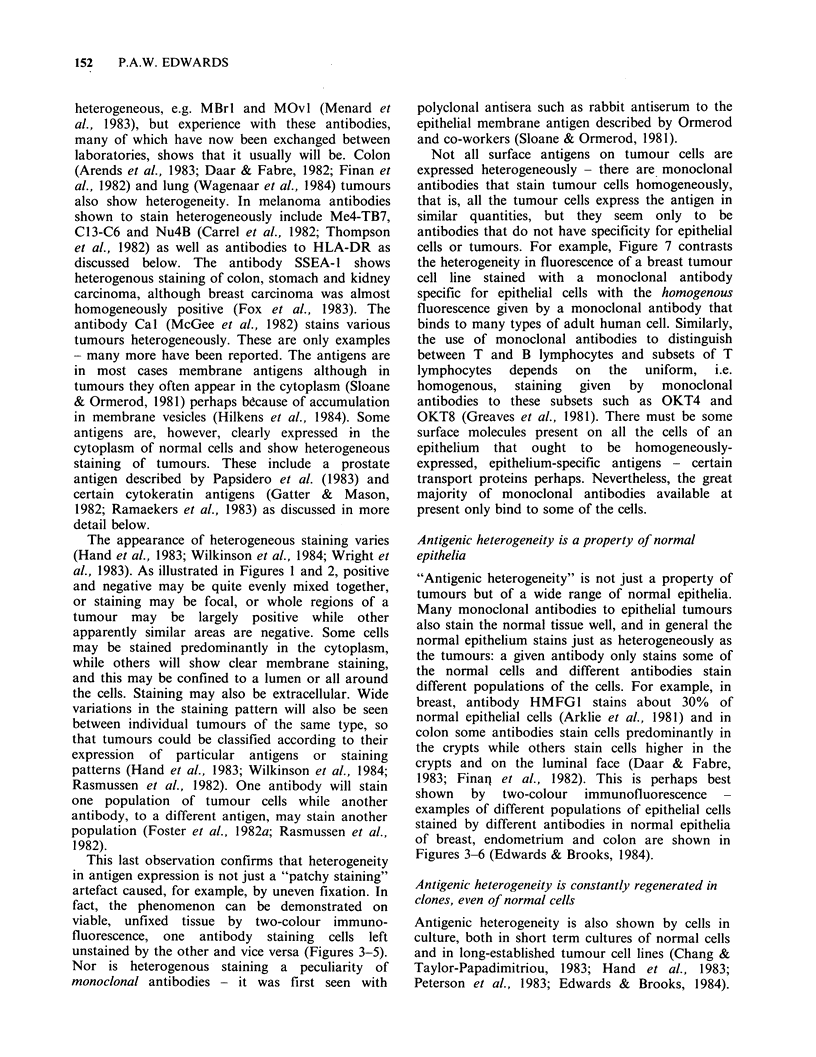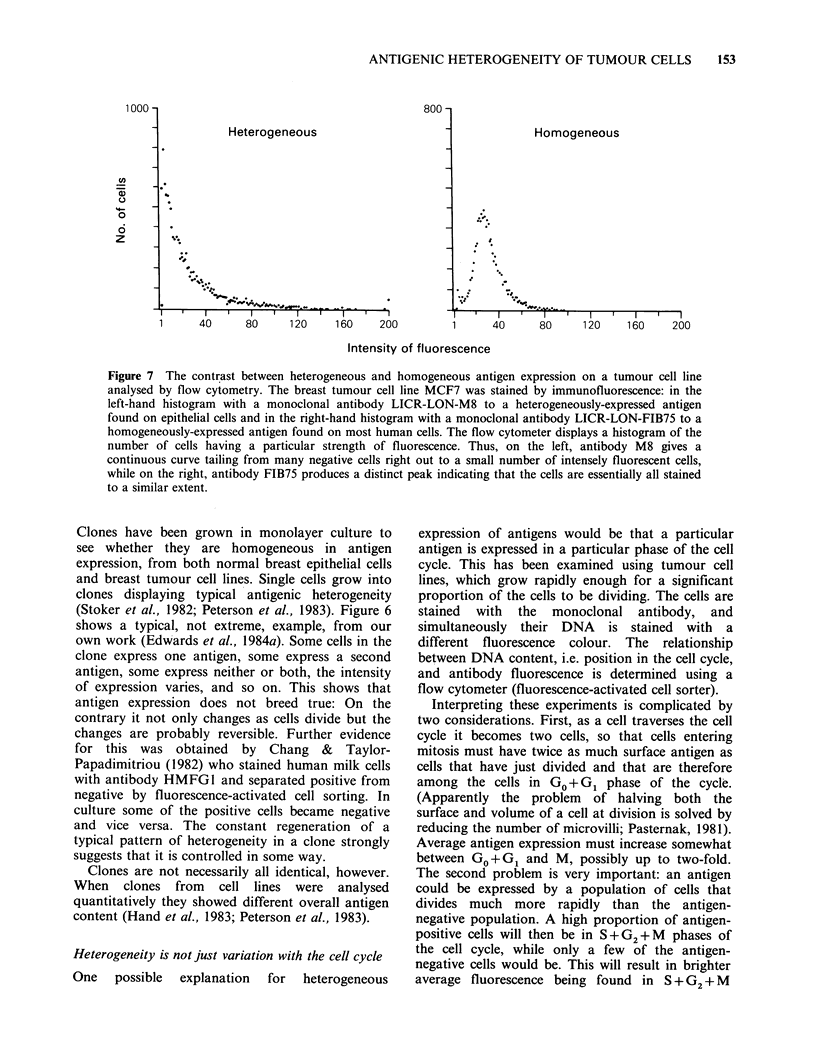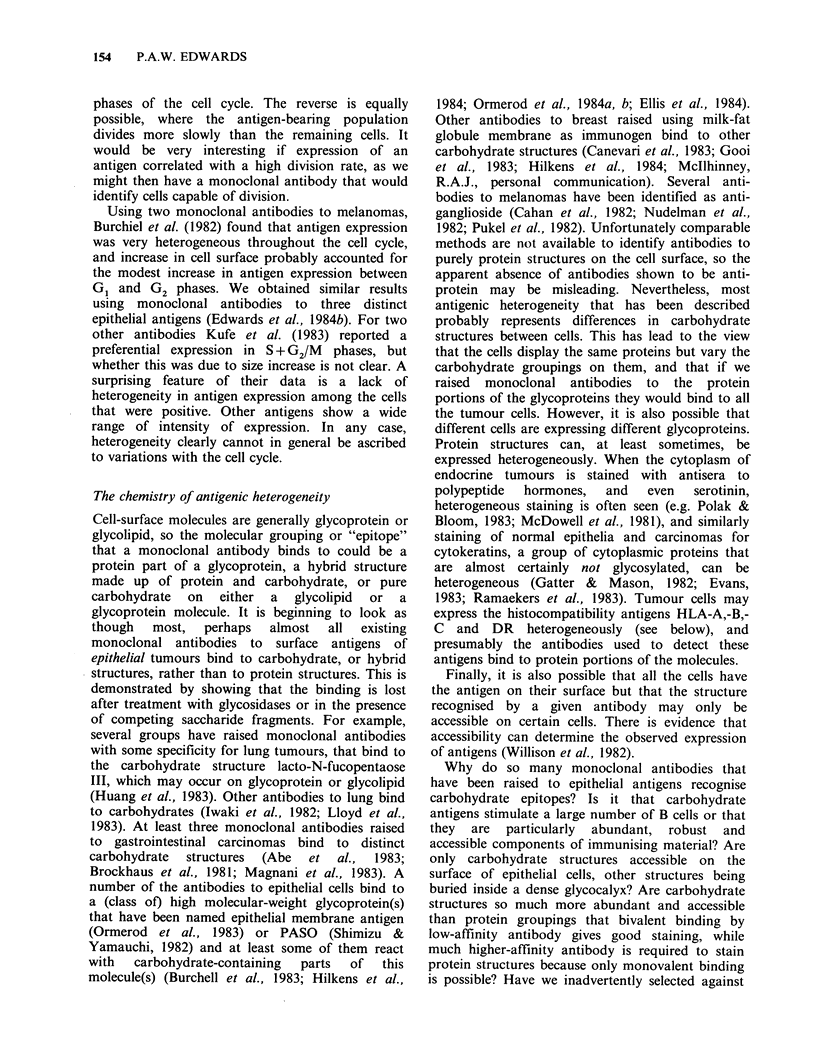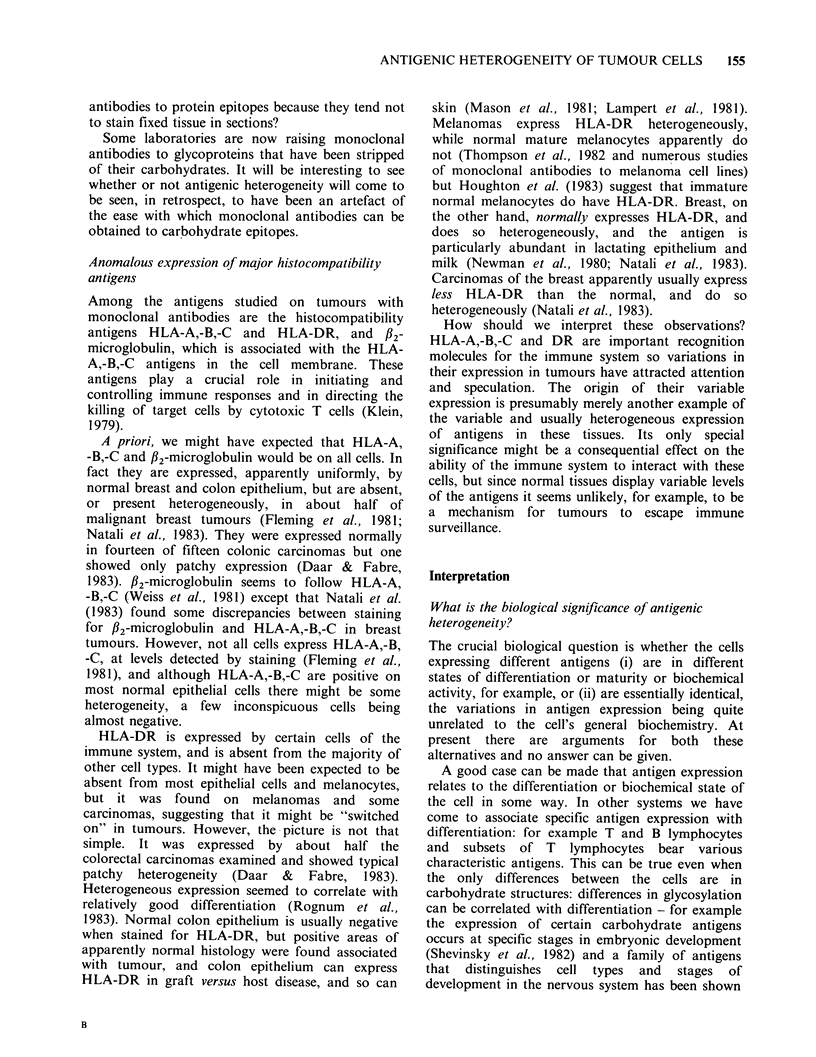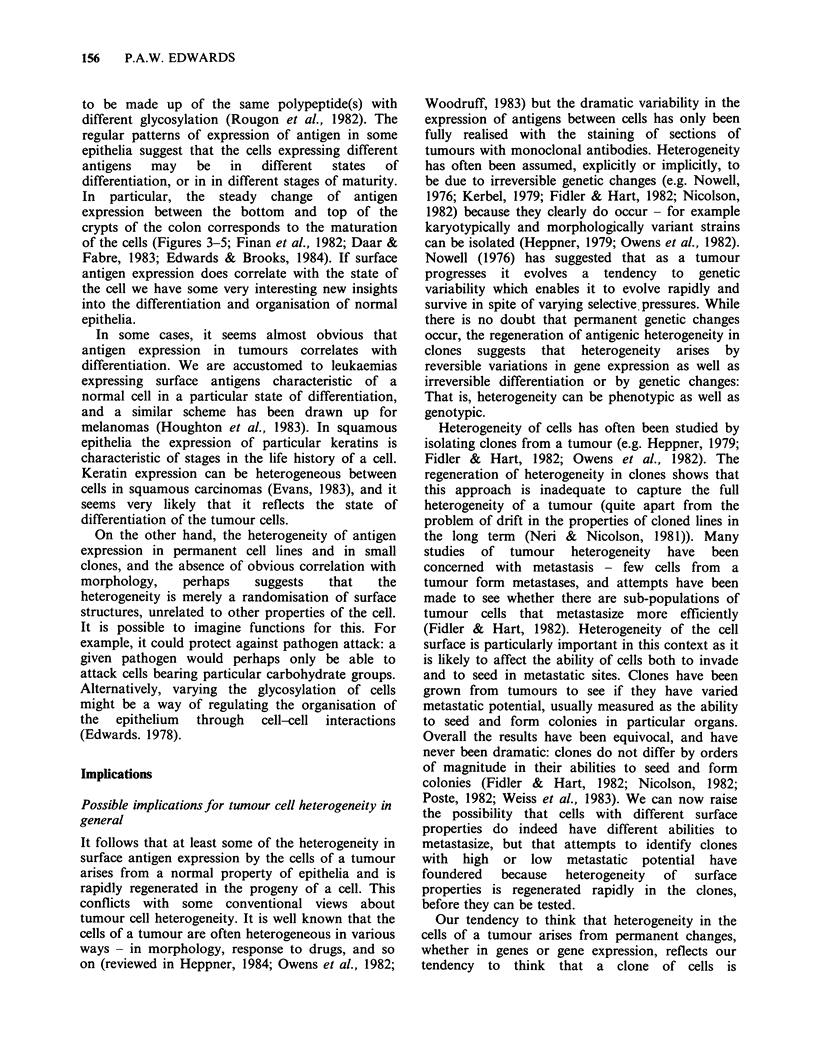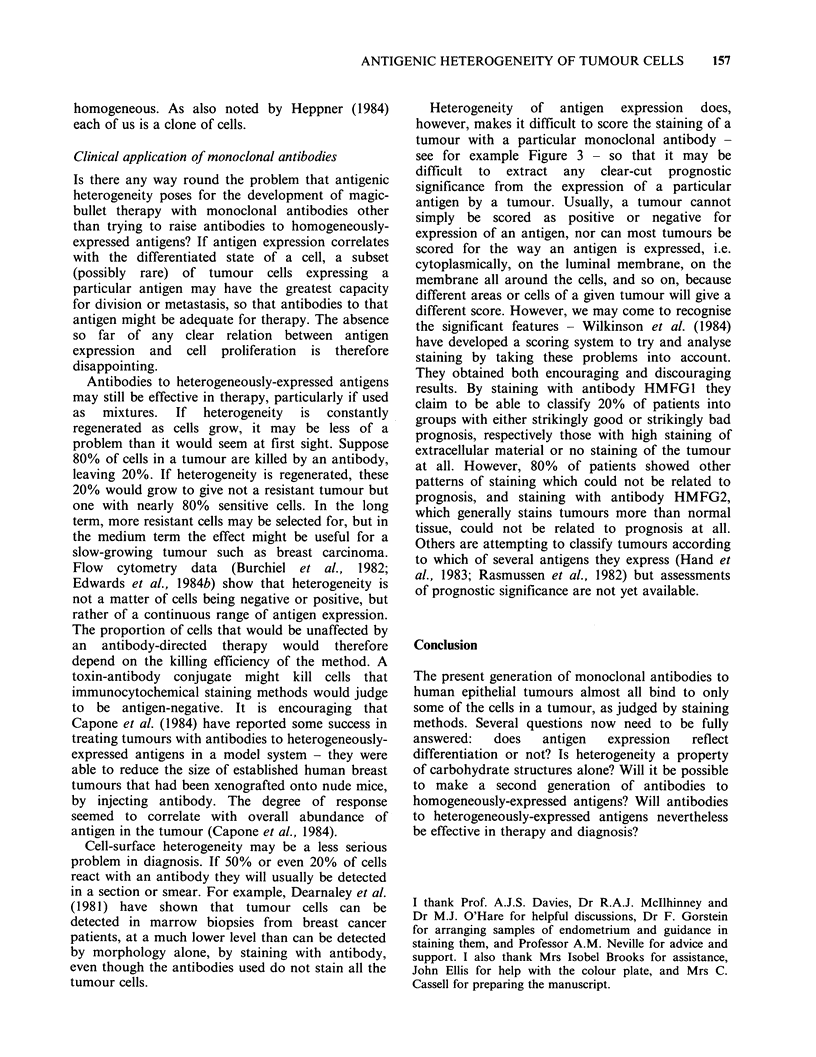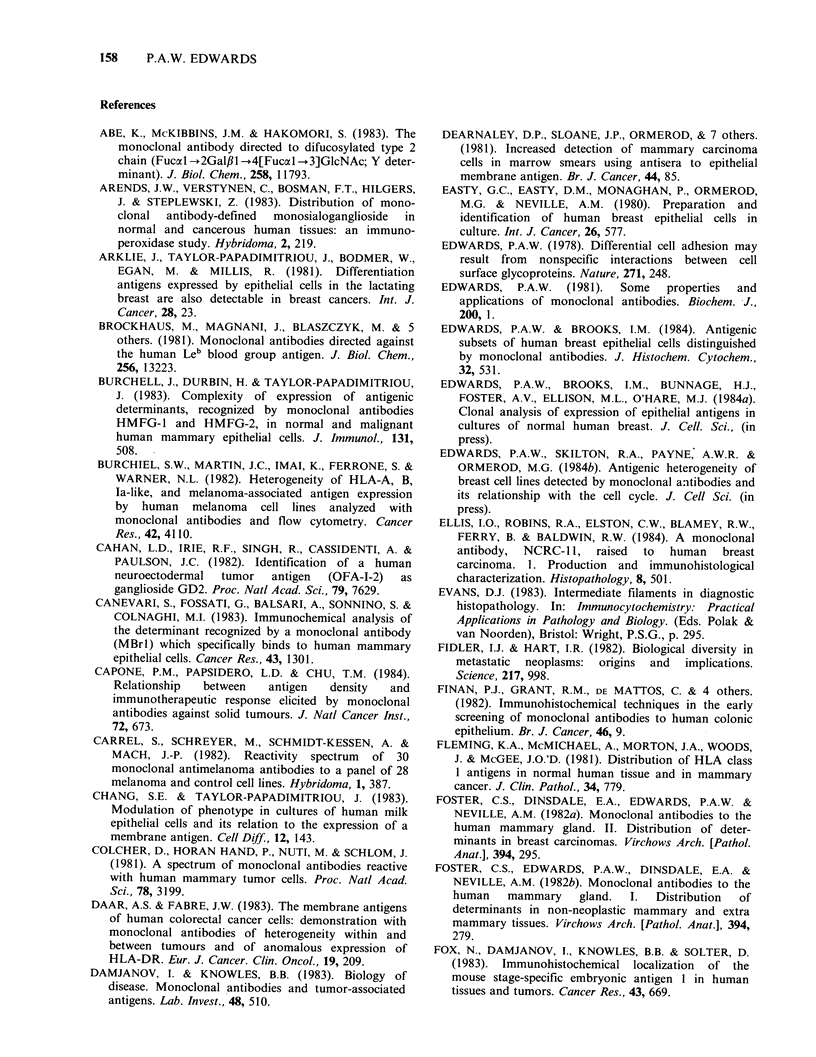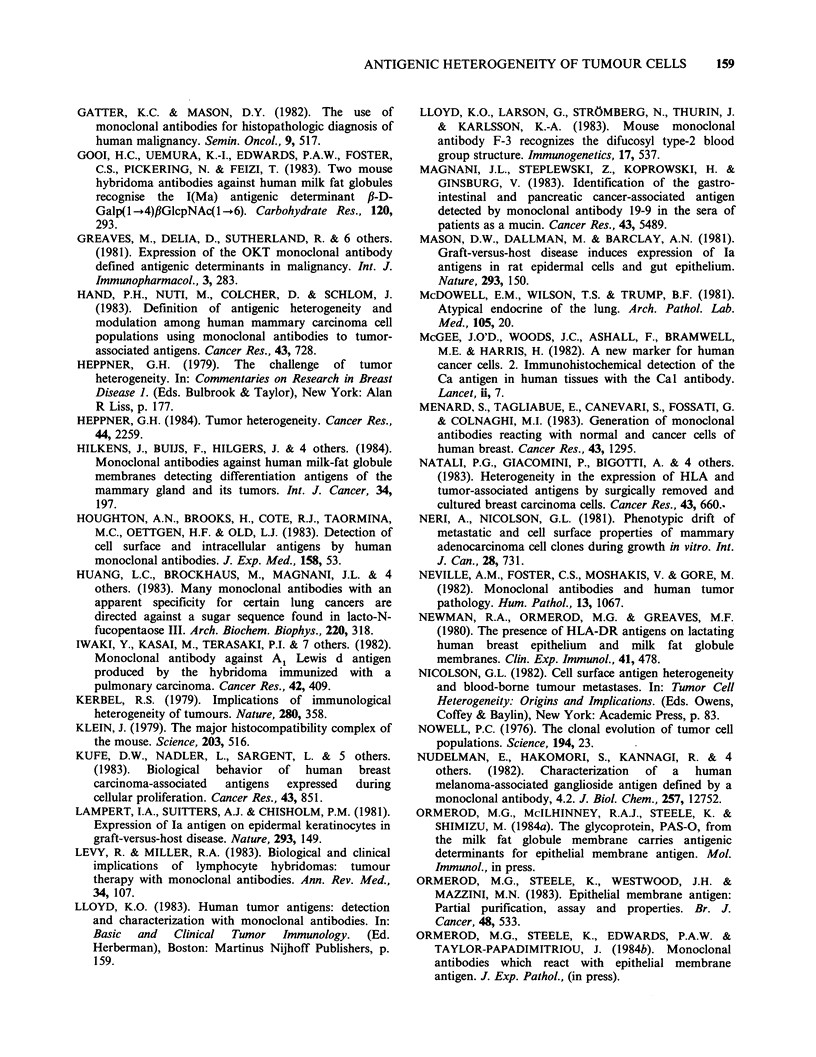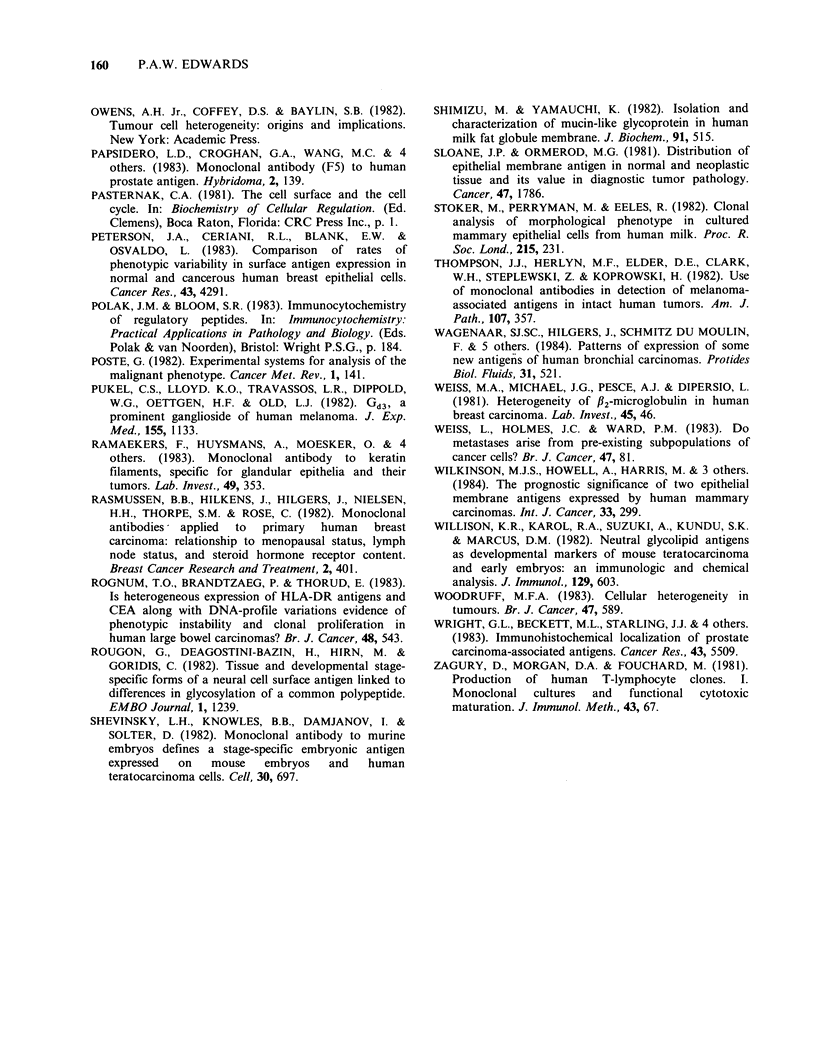# Heterogeneous expression of cell-surface antigens in normal epithelia and their tumours, revealed by monoclonal antibodies.

**DOI:** 10.1038/bjc.1985.24

**Published:** 1985-02

**Authors:** P. A. Edwards

## Abstract

**Images:**


*Br. J. Cancer* (1985), **51**, 149–160

**Review**

### Heterogenous expression of cell-surface antigens in normal epithelia and their tumours, revealed by monoclonal antibodies

P.A.W. Edwards*

*Ludwig Institute for Cancer Research (London Branch), Royal Marsden Hospital, Sutton, Surrey SM2 5PX, UK.*

**Summary** Most monoclonal antibodies that have been raised to human epithelial tumours bind to only some of the cells in a tumour, showing that tumour cells are very heterogenous in their expression of antigens. Normal epithelia show the same heterogeneity of antigen expression, as also do cell lines and clones of epithelial cells in culture. It is not related to the mitotic cell cycle. Many, probably most of the antigenic determinants to which the antibodies bind are carbohydrate structures. It is not clear whether variations in antigen expression reflect variations in the differentiated state of the cells or merely variations in the carbohydrate structures on otherwise identical cells, nor is ir clear whether antibodies could be made that bind to all tumour cells by avoiding antibodies to carbohydrate structures. The normal and apparently reversible nature of this heterogeneity of antigen expression conflicts with conventional views that heterogeneity among cells of a tumour is due to permanent genetic change. The heterogeneity within normal clones suggests that cloning is not an adequate way to study heterogeneity in tumour cells. The implications of heterogeneous expression of antigens within tumours for therapeutic and diagnostic application of antibodies are discussed.

Many laboratories have been raising monoclonal antibodies to cell-surface antigens of human epithelia and epithelial tumours in the hope of applying them to the diagnosis, detection and therapy of malignancy (reviews: monoclonal antibodies in general, Edwards, 1981; used in pathology, Neville *et al.,* 1982; Damjanov & Knowles, 1983; in therapy, Levy & Miller, 1983; antigens on tumour cells, Lloyd, 1983). Perhaps the most interesting observation that has come out of this work is that when a tumour or normal epithelium is stained with a given antibody only some of the cells in the tumour or epithelium bind the antibody. In other words, the antibodies appear to define distinct populations of cells in normal and neoplastic epithelia. This clearly poses problems for therapy and diagnosis, as a given antibody will only bind detectably to a proportion of cells in a given tumour.

In this review I have attempted to summarise what we know and do not know about this phenomenon and its biological significance, and to discuss its implications not only for the development of antibody therapy and diagnosis, but also for the study of the cellular heterogeneity of tumours.

The illustrations are taken from work of this laboratory purely for convenience and the advantages of colour. As far as I am aware the general phenomena of staining that are illustrated are similar to those obtained with the antibodies raised in other laboratories.

### The observations

### *Most monoclonal antibodies to epithelial tumours stain the cells of a tumour heterogeneously*

Typically, when a section through a tumour is stained with a monoclonal antibody, some cells are positive while others of apparently identical morphology are negative. Representative examples are shown in Figures 1 and 2. It seems that the great majority of monoclonal antibodies raised to epithelial tumours behave like this. Of the monoclonal antibodies raised to breast tumours, the majority have been reported to show heterogeneity (Arklie *et al.,* 1981; Colcher *et al.,* 1981; Ellis *et al.,* 1984; Foster *et al.,* 1982*a, b*; Hilkens *et al.,* 1984). Of other anti-breast antibodies that have been tested on sections of tumour, it is not stated whether staining is

*Present Address: Department of Pathology, University of Cambridge, Tennis Court Road, Cambridge, UK.
Received 25 October 1984.

© The Macmillan Press Ltd., 1985

2

*Legend to Colour Plate*

*Examples of heterogeneous antigen expression by tumours, normal epithelium and cloned cells in culture.*

For convenience these examples were obtained using the author's monoclonal antibodies, but they are representative of the antibodies in the field.

**Figures 1 and 2** Typical staining of tumour sections by monoclonal antibodies. Some cells are positive, others, apparently of the same morphology, are negative. In (**1**) the distribution of positive cells is scattered, in (**2**) whole areas of tumour are positive or negative. Conventional paraffin sections were stained by the immunoperoxidase method which produces a brown reaction product where antibody has bound. Blue is a haemalum counterstain to show nuclei. Monoclonal antibody LICR-LON-M8 (Foster *et al.*, 1982*b*) on breast carcinoma. (1) × 200; (2) × 80. (*Courtesy of Dr P. Monaghan and J.D.B. Roberts*).

**Figures 3 to 5** Antigenic heterogeneity in normal epithelia, demonstrated by two-colour immunofluorescence (Edwards & Brooks, 1984 and unpublished). (**3**) Normal endometrium, which displays an apparently random pattern of antigens. The tissue was stained intact and unfixed, as a sheet, and is viewed *en face,* not in section. Each patch of colour is the apical membrane of an individual cell. Monoclonal antibodies LICR-LON-M8 (green fluorescence) and LICR-LON-M24 (red fluorescence). Yellow cells are those stained by both antibodies, × 300. (**4**) Normal breast duct epithelium, which shows reproducible, recognisable patterns of antigen expression. Stained as a sheet of tissue obtained by dissecting out a duct and splitting it along its length (Edwards & Brooks, 1984). Viewed *en face,* not in section, as in (**3**), × 300. Antibodies and colours as in (**3**). (**5**) Frozen section of normal human colon, where antigen expression seems to be linked to the maturation of the cells. Staining by antibody LICR-LON-M8 (in green) seems concentrated in the crypts, and as the cells pass up the crypts they increasingly express antigen LICR-LON-M24 (red). Antibodies and colours as in (**3**) and (**4**), × 70. **Figure 6** Antigenic heterogeneity in a single-cell clone of normal breast epithelial cells, showing that heterogeneity is rapidly regenerated in clones. Normal breast epithelium cells were obtained by digestion with collagenase (Easty *et al.*, 1980) then trypsin + EDTA. Single cells were isolated by micromanipulation to ensure single-cell origin of the clones (Zagury *et al.*, 1981), and grown on a sparse feeder layer in petri dishes (Stoker *et al.*, 1982). Monoclonal antibodies LICR-LON-M8 (green) and LICR-LON-M24 (red), × 200.

3

4

5

6

heterogeneous, e.g. MBr1 and MOv1 (Menard *et al.*, 1983), but experience with these antibodies, many of which have now been exchanged between laboratories, shows that it usually will be. Colon (Arends *et al.*, 1983; Daar & Fabre, 1982; Finan *et al.*, 1982) and lung (Wagenaar *et al.*, 1984) tumours also show heterogeneity. In melanoma antibodies shown to stain heterogeneously include Me4-TB7, C13-C6 and Nu4B (Carrel *et al.*, 1982; Thompson *et al.*, 1982) as well as antibodies to HLA-DR as discussed below. The antibody SSEA-1 shows heterogenous staining of colon, stomach and kidney carcinoma, although breast carcinoma was almost homogeneously positive (Fox *et al.*, 1983). The antibody Ca1 (McGee *et al.*, 1982) stains various tumours heterogeneously. These are only examples – many more have been reported. The antigens are in most cases membrane antigens although in tumours they often appear in the cytoplasm (Sloane & Ormerod, 1981) perhaps because of accumulation in membrane vesicles (Hilkens *et al.*, 1984). Some antigens are, however, clearly expressed in the cytoplasm of normal cells and show heterogeneous staining of tumours. These include a prostate antigen described by Papsidero *et al.* (1983) and certain cytokeratin antigens (Gatter & Mason, 1982; Ramaekers *et al.*, 1983) as discussed in more detail below.

The appearance of heterogeneous staining varies (Hand *et al.*, 1983; Wilkinson *et al.*, 1984; Wright *et al.*, 1983). As illustrated in Figures 1 and 2, positive and negative may be quite evenly mixed together, or staining may be focal, or whole regions of a tumour may be largely positive while other apparently similar areas are negative. Some cells may be stained predominantly in the cytoplasm, while others will show clear membrane staining, and this may be confined to a lumen or all around the cells. Staining may also be extracellular. Wide variations in the staining pattern will also be seen between individual tumours of the same type, so that tumours could be classified according to their expression of particular antigens or staining patterns (Hand *et al.*, 1983; Wilkinson *et al.*, 1984; Rasmussen *et al.*, 1982). One antibody will stain one population of tumour cells while another antibody, to a different antigen, may stain another population (Foster *et al.*, 1982*a*; Rasmussen *et al.*, 1982).

This last observation confirms that heterogeneity in antigen expression is not just a "patchy staining" artefact caused, for example, by uneven fixation. In fact, the phenomenon can be demonstrated on viable, unfixed tissue by two-colour immunofluorescence, one antibody staining cells left unstained by the other and vice versa (Figures 3–5). Nor is heterogenous staining a peculiarity of *monoclonal* antibodies – it was first seen with polyclonal antisera such as rabbit antiserum to the epithelial membrane antigen described by Ormerod and co-workers (Sloane & Ormerod, 1981).

Not all surface antigens on tumour cells are expressed heterogeneously – there are monoclonal antibodies that stain tumour cells homogeneously, that is, all the tumour cells express the antigen in similar quantities, but they seem only to be antibodies that do not have specificity for epithelial cells or tumours. For example, Figure 7 contrasts the heterogeneity in fluorescence of a breast tumour cell line stained with a monoclonal antibody specific for epithelial cells with the *homogenous* fluorescence given by a monoclonal antibody that binds to many types of adult human cell. Similarly, the use of monoclonal antibodies to distinguish between T and B lymphocytes and subsets of T lymphocytes depends on the uniform, i.e. homogenous, staining given by monoclonal antibodies to these subsets such as OKT4 and OKT8 (Greaves *et al.*, 1981). There must be some surface molecules present on all the cells of an epithelium that ought to be homogeneously-expressed, epithelium-specific antigens – certain transport proteins perhaps. Nevertheless, the great majority of monoclonal antibodies available at present only bind to some of the cells.

### *Antigenic heterogeneity is a property of normal epithelia*

"Antigenic heterogeneity" is not just a property of tumours but of a wide range of normal epithelia. Many monoclonal antibodies to epithelial tumours also stain the normal tissue well, and in general the normal epithelium stains just as heterogeneously as the tumours: a given antibody only stains some of the normal cells and different antibodies stain different populations of the cells. For example, in breast, antibody HMFG1 stains about 30% of normal epithelial cells (Arklie *et al.*, 1981) and in colon some antibodies stain cells predominantly in the crypts while others stain cells higher in the crypts and on the luminal face (Daar & Fabre, 1983; Finan *et al.*, 1982). This is perhaps best shown by two-colour immunofluorescence – examples of different populations of epithelial cells stained by different antibodies in normal epithelia of breast, endometrium and colon are shown in Figures 3–6 (Edwards & Brooks, 1984).

### *Antigenic heterogeneity is constantly regenerated in clones, even of normal cells*

Antigenic heterogeneity is also shown by cells in culture, both in short term cultures of normal cells and in long-established tumour cell lines (Chang & Taylor-Papadimitriou, 1983; Hand *et al.*, 1983; Peterson *et al.*, 1983; Edwards & Brooks, 1984).

**Figure 7** The contrast between heterogeneous and homogeneous antigen expression on a tumour cell line analysed by flow cytometry. The breast tumour cell line MCF7 was stained by immunofluorescence: in the left-hand histogram with a monoclonal antibody LICR-LON-M8 to a heterogeneously-expressed antigen found on epithelial cells and in the right-hand histogram with a monoclonal antibody LICR-LON-FIB75 to a homogeneously-expressed antigen found on most human cells. The flow cytometer displays a histogram of the number of cells having a particular strength of fluorescence. Thus, on the left, antibody M8 gives a continuous curve tailing from many negative cells right out to a small number of intensely fluorescent cells, while on the right, antibody FIB75 produces a distinct peak indicating that the cells are essentially all stained to a similar extent.

Clones have been grown in monolayer culture to see whether they are homogeneous in antigen expression, from both normal breast epithelial cells and breast tumour cell lines. Single cells grow into clones displaying typical antigenic heterogeneity (Stoker *et al.*, 1982; Peterson *et al.*, 1983). Figure 6 shows a typical, not extreme, example, from our own work (Edwards *et al.*, 1984*a*). Some cells in the clone express one antigen, some express a second antigen, some express neither or both, the intensity of expression varies, and so on. This shows that antigen expression does not breed true: On the contrary it not only changes as cells divide but the changes are probably reversible. Further evidence for this was obtained by Chang & Taylor-Papadimitriou (1982) who stained human milk cells with antibody HMFG1 and separated positive from negative by fluorescence-activated cell sorting. In culture some of the positive cells became negative and vice versa. The constant regeneration of a typical pattern of heterogeneity in a clone strongly suggests that it is controlled in some way.

Clones are not necessarily all identical, however. When clones from cell lines were analysed quantitatively they showed different overall antigen content (Hand *et al.*, 1983; Peterson *et al.*, 1983).

### *Heterogeneity is not just variation with the cell cycle*

One possible explanation for heterogeneous expression of antigens would be that a particular antigen is expressed in a particular phase of the cell cycle. This has been examined using tumour cell lines, which grow rapidly enough for a significant proportion of the cells to be dividing. The cells are stained with the monoclonal antibody, and simultaneously their DNA is stained with a different fluorescence colour. The relationship between DNA content, i.e. position in the cell cycle, and antibody fluorescence is determined using a flow cytometer (fluorescence-activated cell sorter).

Interpreting these experiments is complicated by two considerations. First, as a cell traverses the cell cycle it becomes two cells, so that cells entering mitosis must have twice as much surface antigen as cells that have just divided and that are therefore among the cells in $G_0+G_1$ phase of the cycle. (Apparently the problem of halving both the surface and volume of a cell at division is solved by reducing the number of microvilli; Pasternak, 1981). Average antigen expression must increase somewhat between $G_0+G_1$ and M, possibly up to two-fold. The second problem is very important: an antigen could be expressed by a population of cells that divides much more rapidly than the antigen-negative population. A high proportion of antigen-positive cells will then be in $S+G_2+M$ phases of the cell cycle, while only a few of the antigen-negative cells would be. This will result in brighter average fluorescence being found in $S+G_2+M$

phases of the cell cycle. The reverse is equally possible, where the antigen-bearing population divides more slowly than the remaining cells. It would be very interesting if expression of an antigen correlated with a high division rate, as we might then have a monoclonal antibody that would identify cells capable of division.

Using two monoclonal antibodies to melanomas, Burchiel *et al.* (1982) found that antigen expression was very heterogeneous throughout the cell cycle, and increase in cell surface probably accounted for the modest increase in antigen expression between $G_1$ and $G_2$ phases. We obtained similar results using monoclonal antibodies to three distinct epithelial antigens (Edwards *et al.*, 1984*b*). For two other antibodies Kufe *et al.* (1983) reported a preferential expression in $S+G_2/M$ phases, but whether this was due to size increase is not clear. A surprising feature of their data is a lack of heterogeneity in antigen expression among the cells that were positive. Other antigens show a wide range of intensity of expression. In any case, heterogeneity clearly cannot in general be ascribed to variations with the cell cycle.

*The chemistry of antigenic heterogeneity*

Cell-surface molecules are generally glycoprotein or glycolipid, so the molecular grouping or "epitope" that a monoclonal antibody binds to could be a protein part of a glycoprotein, a hybrid structure made up of protein and carbohydrate, or pure carbohydrate on either a glycolipid or a glycoprotein molecule. It is beginning to look as though most, perhaps almost all existing monoclonal antibodies to surface antigens of *epithelial* tumours bind to carbohydrate, or hybrid structures, rather than to protein structures. This is demonstrated by showing that the binding is lost after treatment with glycosidases or in the presence of competing saccharide fragments. For example, several groups have raised monoclonal antibodies with some specificity for lung tumours, that bind to the carbohydrate structure lacto-N-fucopentaose III, which may occur on glycoprotein or glycolipid (Huang *et al.*, 1983). Other antibodies to lung bind to carbohydrates (Iwaki *et al.*, 1982; Lloyd *et al.*, 1983). At least three monoclonal antibodies raised to gastrointestinal carcinomas bind to distinct carbohydrate structures (Abe *et al.*, 1983; Brockhaus *et al.*, 1981; Magnani *et al.*, 1983). A number of the antibodies to epithelial cells bind to a (class of) high molecular-weight glycoprotein(s) that have been named epithelial membrane antigen (Ormerod *et al.*, 1983) or PASO (Shimizu & Yamauchi, 1982) and at least some of them react with carbohydrate-containing parts of this molecule(s) (Burchell *et al.*, 1983; Hilkens *et al.*, 1984; Ormerod *et al.*, 1984*a, b*; Ellis *et al.*, 1984). Other antibodies to breast raised using milk-fat globule membrane as immunogen bind to other carbohydrate structures (Canevari *et al.*, 1983; Gooi *et al.*, 1983; Hilkens *et al.*, 1984; McIlhinney, R.A.J., personal communication). Several antibodies to melanomas have been identified as antiganglioside (Cahan *et al.*, 1982; Nudelman *et al.*, 1982; Pukel *et al.*, 1982). Unfortunately comparable methods are not available to identify antibodies to purely protein structures on the cell surface, so the apparent absence of antibodies shown to be antiprotein may be misleading. Nevertheless, most antigenic heterogeneity that has been described probably represents differences in carbohydrate structures between cells. This has lead to the view that the cells display the same proteins but vary the carbohydrate groupings on them, and that if we raised monoclonal antibodies to the protein portions of the glycoproteins they would bind to all the tumour cells. However, it is also possible that different cells are expressing different glycoproteins. Protein structures can, at least sometimes, be expressed heterogeneously. When the cytoplasm of endocrine tumours is stained with antisera to polypeptide hormones, and even serotinin, heterogeneous staining is often seen (e.g. Polak & Bloom, 1983; McDowell *et al.*, 1981), and similarly staining of normal epithelia and carcinomas for cytokeratins, a group of cytoplasmic proteins that are almost certainly *not* glycosylated, can be heterogeneous (Gatter & Mason, 1982; Evans, 1983; Ramaekers *et al.*, 1983). Tumour cells may express the histocompatibility antigens HLA-A,-B,-C and DR heterogeneously (see below), and presumably the antibodies used to detect these antigens bind to protein portions of the molecules.

Finally, it is also possible that all the cells have the antigen on their surface but that the structure recognised by a given antibody may only be accessible on certain cells. There is evidence that accessibility can determine the observed expression of antigens (Willison *et al.*, 1982).

Why do so many monoclonal antibodies that have been raised to epithelial antigens recognise carbohydrate epitopes? Is it that carbohydrate antigens stimulate a large number of B cells or that they are particularly abundant, robust and accessible components of immunising material? Are only carbohydrate structures accessible on the surface of epithelial cells, other structures being buried inside a dense glycocalyx? Are carbohydrate structures so much more abundant and accessible than protein groupings that bivalent binding by low-affinity antibody gives good staining, while much higher-affinity antibody is required to stain protein structures because only monovalent binding is possible? Have we inadvertently selected against antibodies to protein epitopes because they tend not to stain fixed tissue in sections?

Some laboratories are now raising monoclonal antibodies to glycoproteins that have been stripped of their carbohydrates. It will be interesting to see whether or not antigenic heterogeneity will come to be seen, in retrospect, to have been an artefact of the ease with which monoclonal antibodies can be obtained to carbohydrate epitopes.

*Anomalous expression of major histocompatibility antigens*

Among the antigens studied on tumours with monoclonal antibodies are the histocompatibility antigens HLA-A,-B,-C and HLA-DR, and $\beta_2$-microglobulin, which is associated with the HLA-A,-B,-C antigens in the cell membrane. These antigens play a crucial role in initiating and controlling immune responses and in directing the killing of target cells by cytotoxic T cells (Klein, 1979).

*A priori*, we might have expected that HLA-A, -B,-C and $\beta_2$-microglobulin would be on all cells. In fact they are expressed, apparently uniformly, by normal breast and colon epithelium, but are absent, or present heterogeneously, in about half of malignant breast tumours (Fleming *et al.*, 1981; Natali *et al.*, 1983). They were expressed normally in fourteen of fifteen colonic carcinomas but one showed only patchy expression (Daar & Fabre, 1983). $\beta_2$-microglobulin seems to follow HLA-A, -B,-C (Weiss *et al.*, 1981) except that Natali *et al.* (1983) found some discrepancies between staining for $\beta_2$-microglobulin and HLA-A,-B,-C in breast tumours. However, not all cells express HLA-A,-B, -C, at levels detected by staining (Fleming *et al.*, 1981), and although HLA-A,-B,-C are positive on most normal epithelial cells there might be some heterogeneity, a few inconspicuous cells being almost negative.

HLA-DR is expressed by certain cells of the immune system, and is absent from the majority of other cell types. It might have been expected to be absent from most epithelial cells and melanocytes, but it was found on melanomas and some carcinomas, suggesting that it might be "switched on" in tumours. However, the picture is not that simple. It was expressed by about half the colorectal carcinomas examined and showed typical patchy heterogeneity (Daar & Fabre, 1983). Heterogeneous expression seemed to correlate with relatively good differentiation (Rognum *et al.*, 1983). Normal colon epithelium is usually negative when stained for HLA-DR, but positive areas of apparently normal histology were found associated with tumour, and colon epithelium can express HLA-DR in graft *versus* host disease, and so can skin (Mason *et al.*, 1981; Lampert *et al.*, 1981). Melanomas express HLA-DR heterogeneously, while normal mature melanocytes apparently do not (Thompson *et al.*, 1982 and numerous studies of monoclonal antibodies to melanoma cell lines) but Houghton *et al.* (1983) suggest that immature normal melanocytes do have HLA-DR. Breast, on the other hand, *normally* expresses HLA-DR, and does so heterogeneously, and the antigen is particularly abundant in lactating epithelium and milk (Newman *et al.*, 1980; Natali *et al.*, 1983). Carcinomas of the breast apparently usually express *less* HLA-DR than the normal, and do so heterogeneously (Natali *et al.*, 1983).

How should we interpret these observations? HLA-A,-B,-C and DR are important recognition molecules for the immune system so variations in their expression in tumours have attracted attention and speculation. The origin of their variable expression is presumably merely another example of the variable and usually heterogeneous expression of antigens in these tissues. Its only special significance might be a consequential effect on the ability of the immune system to interact with these cells, but since normal tissues display variable levels of the antigens it seems unlikely, for example, to be a mechanism for tumours to escape immune surveillance.

### Interpretation

*What is the biological significance of antigenic heterogeneity?*

The crucial biological question is whether the cells expressing different antigens (i) are in different states of differentiation or maturity or biochemical activity, for example, or (ii) are essentially identical, the variations in antigen expression being quite unrelated to the cell's general biochemistry. At present there are arguments for both these alternatives and no answer can be given.

A good case can be made that antigen expression relates to the differentiation or biochemical state of the cell in some way. In other systems we have come to associate specific antigen expression with differentiation: for example T and B lymphocytes and subsets of T lymphocytes bear various characteristic antigens. This can be true even when the only differences between the cells are in carbohydrate structures: differences in glycosylation can be correlated with differentiation – for example the expression of certain carbohydrate antigens occurs at specific stages in embryonic development (Shevinsky *et al.*, 1982) and a family of antigens that distinguishes cell types and stages of development in the nervous system has been shown to be made up of the same polypeptide(s) with different glycosylation (Rougon *et al.,* 1982). The regular patterns of expression of antigen in some epithelia suggest that the cells expressing different antigens may be in different states of differentiation, or in in different stages of maturity. In particular, the steady change of antigen expression between the bottom and top of the crypts of the colon corresponds to the maturation of the cells (Figures 3–5; Finan *et al.,* 1982; Daar & Fabre, 1983; Edwards & Brooks, 1984). If surface antigen expression does correlate with the state of the cell we have some very interesting new insights into the differentiation and organisation of normal epithelia.

In some cases, it seems almost obvious that antigen expression in tumours correlates with differentiation. We are accustomed to leukaemias expressing surface antigens characteristic of a normal cell in a particular state of differentiation, and a similar scheme has been drawn up for melanomas (Houghton *et al.,* 1983). In squamous epithelia the expression of particular keratins is characteristic of stages in the life history of a cell. Keratin expression can be heterogeneous between cells in squamous carcinomas (Evans, 1983), and it seems very likely that it reflects the state of differentiation of the tumour cells.

On the other hand, the heterogeneity of antigen expression in permanent cell lines and in small clones, and the absence of obvious correlation with morphology, perhaps suggests that the heterogeneity is merely a randomisation of surface structures, unrelated to other properties of the cell. It is possible to imagine functions for this. For example, it could protect against pathogen attack: a given pathogen would perhaps only be able to attack cells bearing particular carbohydrate groups. Alternatively, varying the glycosylation of cells might be a way of regulating the organisation of the epithelium through cell–cell interactions (Edwards. 1978).

### Implications

### *Possible implications for tumour cell heterogeneity in general*

It follows that at least some of the heterogeneity in surface antigen expression by the cells of a tumour arises from a normal property of epithelia and is rapidly regenerated in the progeny of a cell. This conflicts with some conventional views about tumour cell heterogeneity. It is well known that the cells of a tumour are often heterogeneous in various ways – in morphology, response to drugs, and so on (reviewed in Heppner, 1984; Owens *et al.,* 1982; Woodruff, 1983) but the dramatic variability in the expression of antigens between cells has only been fully realised with the staining of sections of tumours with monoclonal antibodies. Heterogeneity has often been assumed, explicitly or implicitly, to be due to irreversible genetic changes (e.g. Nowell, 1976; Kerbel, 1979; Fidler & Hart, 1982; Nicolson, 1982) because they clearly do occur – for example karyotypically and morphologically variant strains can be isolated (Heppner, 1979; Owens *et al.,* 1982). Nowell (1976) has suggested that as a tumour progresses it evolves a tendency to genetic variability which enables it to evolve rapidly and survive in spite of varying selective pressures. While there is no doubt that permanent genetic changes occur, the regeneration of antigenic heterogeneity in clones suggests that heterogeneity arises by reversible variations in gene expression as well as irreversible differentiation or by genetic changes: That is, heterogeneity can be phenotypic as well as genotypic.

Heterogeneity of cells has often been studied by isolating clones from a tumour (e.g. Heppner, 1979; Fidler & Hart, 1982; Owens *et al.,* 1982). The regeneration of heterogeneity in clones shows that this approach is inadequate to capture the full heterogeneity of a tumour (quite apart from the problem of drift in the properties of cloned lines in the long term (Neri & Nicolson, 1981)). Many studies of tumour heterogeneity have been concerned with metastasis – few cells from a tumour form metastases, and attempts have been made to see whether there are sub-populations of tumour cells that metastasize more efficiently (Fidler & Hart, 1982). Heterogeneity of the cell surface is particularly important in this context as it is likely to affect the ability of cells both to invade and to seed in metastatic sites. Clones have been grown from tumours to see if they have varied metastatic potential, usually measured as the ability to seed and form colonies in particular organs. Overall the results have been equivocal, and have never been dramatic: clones do not differ by orders of magnitude in their abilities to seed and form colonies (Fidler & Hart, 1982; Nicolson, 1982; Poste, 1982; Weiss *et al.,* 1983). We can now raise the possibility that cells with different surface properties do indeed have different abilities to metastasize, but that attempts to identify clones with high or low metastatic potential have foundered because heterogeneity of surface properties is regenerated rapidly in the clones, before they can be tested.

Our tendency to think that heterogeneity in the cells of a tumour arises from permanent changes, whether in genes or gene expression, reflects our tendency to think that a clone of cells is homogeneous. As also noted by Heppner (1984) each of us is a clone of cells.

*Clinical application of monoclonal antibodies*

Is there any way round the problem that antigenic heterogeneity poses for the development of magic-bullet therapy with monoclonal antibodies other than trying to raise antibodies to homogeneously-expressed antigens? If antigen expression correlates with the differentiated state of a cell, a subset (possibly rare) of tumour cells expressing a particular antigen may have the greatest capacity for division or metastasis, so that antibodies to that antigen might be adequate for therapy. The absence so far of any clear relation between antigen expression and cell proliferation is therefore disappointing.

Antibodies to heterogeneously-expressed antigens may still be effective in therapy, particularly if used as mixtures. If heterogeneity is constantly regenerated as cells grow, it may be less of a problem than it would seem at first sight. Suppose 80% of cells in a tumour are killed by an antibody, leaving 20%. If heterogeneity is regenerated, these 20% would grow to give not a resistant tumour but one with nearly 80% sensitive cells. In the long term, more resistant cells may be selected for, but in the medium term the effect might be useful for a slow-growing tumour such as breast carcinoma. Flow cytometry data (Burchiel *et al.*, 1982; Edwards *et al.*, 1984*b*) show that heterogeneity is not a matter of cells being negative or positive, but rather of a continuous range of antigen expression. The proportion of cells that would be unaffected by an antibody-directed therapy would therefore depend on the killing efficiency of the method. A toxin-antibody conjugate might kill cells that immunocytochemical staining methods would judge to be antigen-negative. It is encouraging that Capone *et al.* (1984) have reported some success in treating tumours with antibodies to heterogeneously-expressed antigens in a model system – they were able to reduce the size of established human breast tumours that had been xenografted onto nude mice, by injecting antibody. The degree of response seemed to correlate with overall abundance of antigen in the tumour (Capone *et al.*, 1984).

Cell-surface heterogeneity may be a less serious problem in diagnosis. If 50% or even 20% of cells react with an antibody they will usually be detected in a section or smear. For example, Dearnaley *et al.* (1981) have shown that tumour cells can be detected in marrow biopsies from breast cancer patients, at a much lower level than can be detected by morphology alone, by staining with antibody, even though the antibodies used do not stain all the tumour cells.

Heterogeneity of antigen expression does, however, makes it difficult to score the staining of a tumour with a particular monoclonal antibody – see for example Figure 3 – so that it may be difficult to extract any clear-cut prognostic significance from the expression of a particular antigen by a tumour. Usually, a tumour cannot simply be scored as positive or negative for expression of an antigen, nor can most tumours be scored for the way an antigen is expressed, i.e. cytoplasmically, on the luminal membrane, on the membrane all around the cells, and so on, because different areas or cells of a given tumour will give a different score. However, we may come to recognise the significant features – Wilkinson *et al.* (1984) have developed a scoring system to try and analyse staining by taking these problems into account. They obtained both encouraging and discouraging results. By staining with antibody HMFG1 they claim to be able to classify 20% of patients into groups with either strikingly good or strikingly bad prognosis, respectively those with high staining of extracellular material or no staining of the tumour at all. However, 80% of patients showed other patterns of staining which could not be related to prognosis, and staining with antibody HMFG2, which generally stains tumours more than normal tissue, could not be related to prognosis at all. Others are attempting to classify tumours according to which of several antigens they express (Hand *et al.*, 1983; Rasmussen *et al.*, 1982) but assessments of prognostic significance are not yet available.

### Conclusion

The present generation of monoclonal antibodies to human epithelial tumours almost all bind to only some of the cells in a tumour, as judged by staining methods. Several questions now need to be fully answered: does antigen expression reflect differentiation or not? Is heterogeneity a property of carbohydrate structures alone? Will it be possible to make a second generation of antibodies to homogeneously-expressed antigens? Will antibodies to heterogeneously-expressed antigens nevertheless be effective in therapy and diagnosis?

I thank Prof. A.J.S. Davies, Dr R.A.J. McIlhinney and Dr M.J. O'Hare for helpful discussions, Dr F. Gorstein for arranging samples of endometrium and guidance in staining them, and Professor A.M. Neville for advice and support. I also thank Mrs Isobel Brooks for assistance, John Ellis for help with the colour plate, and Mrs C. Cassell for preparing the manuscript.

### References

ABE, K., McKIBBINS, J.M. & HAKOMORI, S. (1983). The monoclonal antibody directed to difucosylated type 2 chain (Fucα1→2Galβ1→4[Fucα1→3]GlcNAc; Y determinant). *J. Biol. Chem.*, **258**, 11793.

ARENDS, J.W., VERSTYNEN, C., BOSMAN, F.T., HILGERS, J. & STEPLEWSKI, Z. (1983). Distribution of monoclonal antibody-defined monosialoganglioside in normal and cancerous human tissues: an immunoperoxidase study. *Hybridoma*, **2**, 219.

ARKLIE, J., TAYLOR-PAPADIMITRIOU, J., BODMER, W., EGAN, M. & MILLIS, R. (1981). Differentiation antigens expressed by epithelial cells in the lactating breast are also detectable in breast cancers. *Int. J. Cancer*, **28**, 23.

BROCKHAUS, M., MAGNANI, J., BLASZCZYK, M. & 5 others. (1981). Monoclonal antibodies directed against the human $Le^b$ blood group antigen. *J. Biol. Chem.*, **256**, 13223.

BURCHELL, J., DURBIN, H. & TAYLOR-PAPADIMITRIOU, J. (1983). Complexity of expression of antigenic determinants, recognized by monoclonal antibodies HMFG-1 and HMFG-2, in normal and malignant human mammary epithelial cells. *J. Immunol.*, **131**, 508.

BURCHIEL, S.W., MARTIN, J.C., IMAI, K., FERRONE, S. & WARNER, N.L. (1982). Heterogeneity of HLA-A, B, Ia-like, and melanoma-associated antigen expression by human melanoma cell lines analyzed with monoclonal antibodies and flow cytometry. *Cancer Res.*, **42**, 4110.

CAHAN, L.D., IRIE, R.F., SINGH, R., CASSIDENTI, A. & PAULSON, J.C. (1982). Identification of a human neuroectodermal tumor antigen (OFA-I-2) as ganglioside GD2. *Proc. Natl Acad. Sci.*, **79**, 7629.

CANEVARI, S., FOSSATI, G., BALSARI, A., SONNINO, S. & COLNAGHI, M.I. (1983). Immunochemical analysis of the determinant recognized by a monoclonal antibody (MBr1) which specifically binds to human mammary epithelial cells. *Cancer Res.*, **43**, 1301.

CAPONE, P.M., PAPSIDERO, L.D. & CHU, T.M. (1984). Relationship between antigen density and immunotherapeutic response elicited by monoclonal antibodies against solid tumours. *J. Natl Cancer Inst.*, **72**, 673.

CARREL, S., SCHREYER, M., SCHMIDT-KESSEN, A. & MACH, J.-P. (1982). Reactivity spectrum of 30 monoclonal antimelanoma antibodies to a panel of 28 melanoma and control cell lines. *Hybridoma*, **1**, 387.

CHANG, S.E. & TAYLOR-PAPADIMITRIOU, J. (1983). Modulation of phenotype in cultures of human milk epithelial cells and its relation to the expression of a membrane antigen. *Cell Diff.*, **12**, 143.

COLCHER, D., HORAN HAND, P., NUTI, M. & SCHLOM, J. (1981). A spectrum of monoclonal antibodies reactive with human mammary tumor cells. *Proc. Natl Acad. Sci.*, **78**, 3199.

DAAR, A.S. & FABRE, J.W. (1983). The membrane antigens of human colorectal cancer cells: demonstration with monoclonal antibodies of heterogeneity within and between tumours and of anomalous expression of HLA-DR. *Eur. J. Cancer. Clin. Oncol.*, **19**, 209.

DAMJANOV, I. & KNOWLES, B.B. (1983). Biology of disease. Monoclonal antibodies and tumor-associated antigens. *Lab. Invest.*, **48**, 510.

DEARNALEY, D.P., SLOANE, J.P., ORMEROD, & 7 others. (1981). Increased detection of mammary carcinoma cells in marrow smears using antisera to epithelial membrane antigen. *Br. J. Cancer*, **44**, 85.

EASTY, G.C., EASTY, D.M., MONAGHAN, P., ORMEROD, M.G. & NEVILLE, A.M. (1980). Preparation and identification of human breast epithelial cells in culture. *Int. J. Cancer*, **26**, 577.

EDWARDS, P.A.W. (1978). Differential cell adhesion may result from nonspecific interactions between cell surface glycoproteins. *Nature*, **271**, 248.

EDWARDS, P.A.W. (1981). Some properties and applications of monoclonal antibodies. *Biochem. J.*, **200**, 1.

EDWARDS, P.A.W. & BROOKS, I.M. (1984). Antigenic subsets of human breast epithelial cells distinguished by monoclonal antibodies. *J. Histochem. Cytochem.*, **32**, 531.

EDWARDS, P.A.W., BROOKS, I.M., BUNNAGE, H.J., FOSTER, A.V., ELLISON, M.L., O'HARE, M.J. (1984*a*). Clonal analysis of expression of epithelial antigens in cultures of normal human breast. *J. Cell. Sci.*, (in press).

EDWARDS, P.A.W., SKILTON, R.A., PAYNE, A.W.R. & ORMEROD, M.G. (1984*b*). Antigenic heterogeneity of breast cell lines detected by monoclonal antibodies and its relationship with the cell cycle. *J. Cell Sci.* (in press).

ELLIS, I.O., ROBINS, R.A., ELSTON, C.W., BLAMEY, R.W., FERRY, B. & BALDWIN, R.W. (1984). A monoclonal antibody, NCRC-11, raised to human breast carcinoma. 1. Production and immunohistological characterization. *Histopathology*, **8**, 501.

EVANS, D.J. (1983). Intermediate filaments in diagnostic histopathology. In: *Immunocytochemistry: Practical Applications in Pathology and Biology*. (Eds. Polak & van Noorden), Bristol: Wright, P.S.G., p. 295.

FIDLER, I.J. & HART, I.R. (1982). Biological diversity in metastatic neoplasms: origins and implications. *Science*, **217**, 998.

FINAN, P.J., GRANT, R.M., DE MATTOS, C. & 4 others. (1982). Immunohistochemical techniques in the early screening of monoclonal antibodies to human colonic epithelium. *Br. J. Cancer*, **46**, 9.

FLEMING, K.A., McMICHAEL, A., MORTON, J.A., WOODS, J. & McGEE, J.O.'D. (1981). Distribution of HLA class 1 antigens in normal human tissue and in mammary cancer. *J. Clin. Pathol.*, **34**, 779.

FOSTER, C.S., DINSDALE, E.A., EDWARDS, P.A.W. & NEVILLE, A.M. (1982*a*). Monoclonal antibodies to the human mammary gland. II. Distribution of determinants in breast carcinomas. *Virchows Arch. [Pathol. Anat.]*, **394**, 295.

FOSTER, C.S., EDWARDS, P.A.W., DINSDALE, E.A. & NEVILLE, A.M. (1982*b*). Monoclonal antibodies to the human mammary gland. I. Distribution of determinants in non-neoplastic mammary and extra mammary tissues. *Virchows Arch. [Pathol. Anat.]*, **394**, 279.

FOX, N., DAMJANOV, I., KNOWLES, B.B. & SOLTER, D. (1983). Immunohistochemical localization of the mouse stage-specific embryonic antigen 1 in human tissues and tumors. *Cancer Res.*, **43**, 669.

GATTER, K.C. & MASON, D.Y. (1982). The use of monoclonal antibodies for histopathologic diagnosis of human malignancy. *Semin. Oncol.*, **9**, 517.

GOOI, H.C., UEMURA, K.-I., EDWARDS, P.A.W., FOSTER, C.S., PICKERING, N. & FEIZI, T. (1983). Two mouse hybridoma antibodies against human milk fat globules recognise the I(Ma) antigenic determinant $\beta$-D-Galp(1→4)$\beta$GlcpNAc(1→6). *Carbohydrate Res.*, **120**, 293.

GREAVES, M., DELIA, D., SUTHERLAND, R. & 6 others. (1981). Expression of the OKT monoclonal antibody defined antigenic determinants in malignancy. *Int. J. Immunopharmacol.*, **3**, 283.

HAND, P.H., NUTI, M., COLCHER, D. & SCHLOM, J. (1983). Definition of antigenic heterogeneity and modulation among human mammary carcinoma cell populations using monoclonal antibodies to tumor-associated antigens. *Cancer Res.*, **43**, 728.

HEPPNER, G.H. (1979). The challenge of tumor heterogeneity. In: *Commentaries on Research in Breast Disease 1*. (Eds. Bulbrook & Taylor), New York: Alan R Liss, p. 177.

HEPPNER, G.H. (1984). Tumor heterogeneity. *Cancer Res.*, **44**, 2259.

HILKENS, J., BUIJS, F., HILGERS, J. & 4 others. (1984). Monoclonal antibodies against human milk-fat globule membranes detecting differentiation antigens of the mammary gland and its tumors. *Int. J. Cancer*, **34**, 197.

HOUGHTON, A.N., BROOKS, H., COTE, R.J., TAORMINA, M.C., OETTGEN, H.F. & OLD, L.J. (1983). Detection of cell surface and intracellular antigens by human monoclonal antibodies. *J. Exp. Med.*, **158**, 53.

HUANG, L.C., BROCKHAUS, M., MAGNANI, J.L. & 4 others. (1983). Many monoclonal antibodies with an apparent specificity for certain lung cancers are directed against a sugar sequence found in lacto-N-fucopentaose III. *Arch. Biochem. Biophys.*, **220**, 318.

IWAKI, Y., KASAI, M., TERASAKI, P.I. & 7 others. (1982). Monoclonal antibody against $A_1$ Lewis d antigen produced by the hybridoma immunized with a pulmonary carcinoma. *Cancer Res.*, **42**, 409.

KERBEL, R.S. (1979). Implications of immunological heterogeneity of tumours. *Nature*, **280**, 358.

KLEIN, J. (1979). The major histocompatibility complex of the mouse. *Science*, **203**, 516.

KUFE, D.W., NADLER, L., SARGENT, L. & 5 others. (1983). Biological behavior of human breast carcinoma-associated antigens expressed during cellular proliferation. *Cancer Res.*, **43**, 851.

LAMPERT, I.A., SUITTERS, A.J. & CHISHOLM, P.M. (1981). Expression of Ia antigen on epidermal keratinocytes in graft-versus-host disease. *Nature*, **293**, 149.

LEVY, R. & MILLER, R.A. (1983). Biological and clinical implications of lymphocyte hybridomas: tumour therapy with monoclonal antibodies. *Ann. Rev. Med.*, **34**, 107.

LLOYD, K.O. (1983). Human tumor antigens: detection and characterization with monoclonal antibodies. In: *Basic and Clinical Tumor Immunology*. (Ed. Herberman), Boston: Martinus Nijhoff Publishers, p. 159.

LLOYD, K.O., LARSON, G., STRÖMBERG, N., THURIN, J. & KARLSSON, K.-A. (1983). Mouse monoclonal antibody F-3 recognizes the difucosyl type-2 blood group structure. *Immunogenetics*, **17**, 537.

MAGNANI, J.L., STEPLEWSKI, Z., KOPROWSKI, H. & GINSBURG, V. (1983). Identification of the gastrointestinal and pancreatic cancer-associated antigen detected by monoclonal antibody 19-9 in the sera of patients as a mucin. *Cancer Res.*, **43**, 5489.

MASON, D.W., DALLMAN, M. & BARCLAY, A.N. (1981). Graft-versus-host disease induces expression of Ia antigens in rat epidermal cells and gut epithelium. *Nature*, **293**, 150.

McDOWELL, E.M., WILSON, T.S. & TRUMP, B.F. (1981). Atypical endocrine of the lung. *Arch. Pathol. Lab. Med.*, **105**, 20.

McGEE, J.O'D., WOODS, J.C., ASHALL, F., BRAMWELL, M.E. & HARRIS, H. (1982). A new marker for human cancer cells. 2. Immunohistochemical detection of the Ca antigen in human tissues with the Ca1 antibody. *Lancet*, **ii**, 7.

MENARD, S., TAGLIABUE, E., CANEVARI, S., FOSSATI, G. & COLNAGHI, M.I. (1983). Generation of monoclonal antibodies reacting with normal and cancer cells of human breast. *Cancer Res.*, **43**, 1295.

NATALI, P.G., GIACOMINI, P., BIGOTTI, A. & 4 others. (1983). Heterogeneity in the expression of HLA and tumor-associated antigens by surgically removed and cultured breast carcinoma cells. *Cancer Res.*, **43**, 660.

NERI, A., NICOLSON, G.L. (1981). Phenotypic drift of metastatic and cell surface properties of mammary adenocarcinoma cell clones during growth *in vitro*. *Int. J. Can.*, **28**, 731.

NEVILLE, A.M., FOSTER, C.S., MOSHAKIS, V. & GORE, M. (1982). Monoclonal antibodies and human tumor pathology. *Hum. Pathol.*, **13**, 1067.

NEWMAN, R.A., ORMEROD, M.G. & GREAVES, M.F. (1980). The presence of HLA-DR antigens on lactating human breast epithelium and milk fat globule membranes. *Clin. Exp. Immunol.*, **41**, 478.

NICOLSON, G.L. (1982). Cell surface antigen heterogeneity and blood-borne tumour metastases. In: *Tumor Cell Heterogeneity: Origins and Implications*. (Eds. Owens, Coffey & Baylin), New York: Academic Press, p. 83.

NOWELL, P.C. (1976). The clonal evolution of tumor cell populations. *Science*, **194**, 23.

NUDELMAN, E., HAKOMORI, S., KANNAGI, R. & 4 others. (1982). Characterization of a human melanoma-associated ganglioside antigen defined by a monoclonal antibody, 4.2. *J. Biol. Chem.*, **257**, 12752.

ORMEROD, M.G., McILHINNEY, R.A.J., STEELE, K. & SHIMIZU, M. (1984*a*). The glycoprotein, PAS-O, from the milk fat globule membrane carries antigenic determinants for epithelial membrane antigen. *Mol. Immunol.*, in press.

ORMEROD, M.G., STEELE, K., WESTWOOD, J.H. & MAZZINI, M.N. (1983). Epithelial membrane antigen: Partial purification, assay and properties. *Br. J. Cancer*, **48**, 533.

ORMEROD, M.G., STEELE, K., EDWARDS, P.A.W. & TAYLOR-PAPADIMITRIOU, J. (1984*b*). Monoclonal antibodies which react with epithelial membrane antigen. *J. Exp. Pathol.*, (in press).

OWENS, A.H. Jr., COFFEY, D.S. & BAYLIN, S.B. (1982). Tumour cell heterogeneity: origins and implications. New York: Academic Press.

PAPSIDERO, L.D., CROGHAN, G.A., WANG, M.C. & 4 others. (1983). Monoclonal antibody (F5) to human prostate antigen. *Hybridoma*, **2**, 139.

PASTERNAK, C.A. (1981). The cell surface and the cell cycle. In: *Biochemistry of Cellular Regulation*. (Ed. Clemens), Boca Raton, Florida: CRC Press Inc., p. 1.

PETERSON, J.A., CERIANI, R.L., BLANK, E.W. & OSVALDO, L. (1983). Comparison of rates of phenotypic variability in surface antigen expression in normal and cancerous human breast epithelial cells. *Cancer Res.*, **43**, 4291.

POLAK, J.M. & BLOOM, S.R. (1983). Immunocytochemistry of regulatory peptides. In: *Immunocytochemistry: Practical Applications in Pathology and Biology*. (Eds. Polak & van Noorden), Bristol: Wright P.S.G., p. 184.

POSTE, G. (1982). Experimental systems for analysis of the malignant phenotype. *Cancer Met. Rev.*, **1**, 141.

PUKEL, C.S., LLOYD. K.O., TRAVASSOS, L.R., DIPPOLD, W.G., OETTGEN, H.F. & OLD, L.J. (1982). $G_{d3}$, a prominent ganglioside of human melanoma. *J. Exp. Med.*, **155**, 1133.

RAMAEKERS, F., HUYSMANS, A., MOESKER, O. & 4 others. (1983). Monoclonal antibody to keratin filaments, specific for glandular epithelia and their tumors. *Lab. Invest.*, **49**, 353.

RASMUSSEN, B.B., HILKENS, J., HILGERS, J., NIELSEN, H.H., THORPE, S.M. & ROSE, C. (1982). Monoclonal antibodies applied to primary human breast carcinoma: relationship to menopausal status, lymph node status, and steroid hormone receptor content. *Breast Cancer Research and Treatment*, **2**, 401.

ROGNUM, T.O., BRANDTZAEG, P. & THORUD, E. (1983). Is heterogeneous expression of HLA-DR antigens and CEA along with DNA-profile variations evidence of phenotypic instability and clonal proliferation in human large bowel carcinomas? *Br. J. Cancer*, **48**, 543.

ROUGON, G., DEAGOSTINI-BAZIN, H., HIRN, M. & GORIDIS, C. (1982). Tissue and developmental stage-specific forms of a neural cell surface antigen linked to differences in glycosylation of a common polypeptide. *EMBO Journal*, **1**, 1239.

SHEVINSKY, L.H., KNOWLES, B.B., DAMJANOV, I. & SOLTER, D. (1982). Monoclonal antibody to murine embryos defines a stage-specific embryonic antigen expressed on mouse embryos and human teratocarcinoma cells. *Cell*, **30**, 697.

SHIMIZU, M. & YAMAUCHI, K. (1982). Isolation and characterization of mucin-like glycoprotein in human milk fat globule membrane. *J. Biochem.*, **91**, 515.

SLOANE, J.P. & ORMEROD, M.G. (1981). Distribution of epithelial membrane antigen in normal and neoplastic tissue and its value in diagnostic tumor pathology. *Cancer*, **47**, 1786.

STOKER, M., PERRYMAN, M. & EELES, R. (1982). Clonal analysis of morphological phenotype in cultured mammary epithelial cells from human milk. *Proc. R. Soc. Lond.*, **215**, 231.

THOMPSON, J.J., HERLYN, M.F., ELDER, D.E., CLARK, W.H., STEPLEWSKI, Z. & KOPROWSKI, H. (1982). Use of monoclonal antibodies in detection of melanoma-associated antigens in intact human tumors. *Am. J. Path.*, **107**, 357.

WAGENAAR, SJ.SC., HILGERS, J., SCHMITZ DU MOULIN, F. & 5 others. (1984). Patterns of expression of some new antigens of human bronchial carcinomas. *Protides Biol. Fluids*, **31**, 521.

WEISS, M.A., MICHAEL, J.G., PESCE, A.J. & DIPERSIO, L. (1981). Heterogeneity of $\beta_2$-microglobulin in human breast carcinoma. *Lab. Invest.*, **45**, 46.

WEISS, L., HOLMES, J.C. & WARD, P.M. (1983). Do metastases arise from pre-existing subpopulations of cancer cells? *Br. J. Cancer*, **47**, 81.

WILKINSON, M.J.S., HOWELL, A., HARRIS, M. & 3 others. (1984). The prognostic significance of two epithelial membrane antigens expressed by human mammary carcinomas. *Int. J. Cancer*, **33**, 299.

WILLISON, K.R., KAROL, R.A., SUZUKI, A., KUNDU, S.K. & MARCUS, D.M. (1982). Neutral glycolipid antigens as developmental markers of mouse teratocarcinoma and early embryos: an immunologic and chemical analysis. *J. Immunol.*, **129**, 603.

WOODRUFF, M.F.A. (1983). Cellular heterogeneity in tumours. *Br. J. Cancer*, **47**, 589.

WRIGHT, G.L., BECKETT, M.L., STARLING, J.J. & 4 others. (1983). Immunohistochemical localization of prostate carcinoma-associated antigens. *Cancer Res.*, **43**, 5509.

ZAGURY, D., MORGAN, D.A. & FOUCHARD, M. (1981). Production of human T-lymphocyte clones. I. Monoclonal cultures and functional cytotoxic maturation. *J. Immunol. Meth.*, **43**, 67.